# The effect of core decompression on local expression of BMP-2, PPAR-γ and bone regeneration in the steroid-induced femoral head osteonecrosis

**DOI:** 10.1186/1471-2474-13-142

**Published:** 2012-08-09

**Authors:** Wei Wang, Liying Liu, Xiaoqian Dang, Shuqiang Ma, Mingyu Zhang, Kunzheng Wang

**Affiliations:** 1 Department of Orthopaedics, Second Affiliated Hospital, Xi’an Jiaotong University School of Medicine, Xi’an, Shaanxi, 710004, People's Republic of China; 2 Key Laboratory of Environment and Genes Related to Diseases, Ministry of Education, Xi’an, 710061, P.R. China; 3 Futian District Hospital, Shenzhen, Guangdong, 518000, People's Republic of China; 4 Xi’an Red Cross Hospital, Xi’an, Shaanxi, 710054, People's Republic of China

**Keywords:** Steroid-induced osteonecrosis, Core decompression, BMP-2, PPAR-γ

## Abstract

**Background:**

To investigate the efficacy of the sole core decompression surgery for the treatment of steroid-induced femoral head osteonecrosis.

**Methods:**

The model was established by administration of steroids in combination with horse serum. The rabbits with bilateral femoral head osteonecrosis were randomly selected to do the one side of core decompression. The other side was used as the sham. Quantitative RT-PCR and western blot techniques were used to measure the local expression of BMP-2 and PPAR-γ. Bone tissues from control and operation groups were histologically analyzed by H&E staining. The comparisons of the local expression of BMP-2 and PPAR-γ and the bone regeneration were further analyzed between different groups at each time point.

**Results:**

The expression of BMP-2 in the osteonecrosis femoral head with or without decompression was significantly lower than that in normal animals. BMP-2 expression both showed the decreasing trend with the increased post-operation time. No significant difference of BMP-2 expression occurred between femoral head osteonecrosis with and without decompression. The PPAR-γ expression in the femoral head osteonecrosis with and without core decompression both was significantly higher than that in control. Its expression pattern showed a significantly increased trend with increased the post-operation time. However, there was no significant difference of PPAR-γ expression between the femoral head osteonecrosis with and without decompression at each time point. Histopathological analysis revealed that new trabecular bone and a large number of osteoblasts were observed in the steroid-induced femoral head osteonecrosis with lateral decompression at 8 weeks after surgery, but there still existed trabecular bone fractures and bone necrosis.

**Conclusions:**

Although decompression takes partial effect in promoting bone regeneration in the early treatment of femoral head osteonecrosis, such an effect does not significantly improve or reverse the pathological changes of femoral head necrosis. Thus, the long-term effect of core decompression in the treatment of steroid-induced femoral head osteonecrosis is not satisfactory.

## Background

Osteonecrosis of the femoral head (ONFH) is a relatively common disease. Tow etiologic factors of this disease are idiopathic and secondary to an underlying systemic disease such as trauma, steroid intake, excessive alcohol use, systemic lupus erythematosus, hemoglobinopathies and dysbarism. Since most of the patients are young individuals, huge social and family burden will come [1] and a consider able number of these patients will need revision surgeries in future. Once osteonecrosis begins, 80 % of the femoral heads will collapse, if no treatment. Glucocorticoids those are commonly prescribed to patients with systemic lupus erythematosus, nephrotic syndrome, and renal transplantation [2], or autoimmune inflammatory diseases, neoplastic diseases organ transplantation have been a leading cause of nontraumatic femoral head osteonecrosis (FHON) [3].

Since the pathogenesis and aetiology of nontraumatic osteonecrosis of the femoral head (ONFH) has not been revealed completely, current treatment of ONFH simply focuses on preventing irreversible complications, namely, biomechanical collapse of the femoral head and osteoarthritis of the hip joint . For the early treatment of osteonecrosis, core decompression is generally considered to slow the natural process of osteonecrosis [4]. Nevertheless its efficacy still remains controversial. Many studies showed that core decompression was performed in the early stage in many cases failed to prevent the progressive collapse. Meanwhile studies displayed that the effect of the core decompression surgery for the treatment of steroid-associated osteonecrosis of femoral head (GONFH) in the severe bone necrosis was not as good as that in the treatment of femoral head osteonecrosis caused by other risk factors.

In steroid-induced ONFH, the Increase of intraosseous pressure due to the elevating of adipogenesis and fat cell hypertrophy in the bone marrow. Although, conventional core decompression also achieves reduction of intra-medullary pressure and provided some pain relief to some extent. It is unable to restrict bone marrow adipogenesis and fat cell hypertrophy, so its efficacy for prevention of steroid-induced ONFH is not ideal.

Recently innovative therapeutic approaches such as the application of mesenchymal and bone marrow mononuclear stem cells (MSCs or BMCs) [5-8], endothelial progenitor cell (EPC)combined [9]. or growth factors (for example bone morphogenetic proteins) with the classical decompression surgery. Other surgical strategies include bone grafting (autologous bone grafts from iliac crest or tibia, allograft cancellous grafts, non-vascularized cortical grafts [10], or tantalum implants [11]), or intertrochanteric de-rotating or Xexion/extension osteotomies. None of these surgical options was found to be superior to any other treatment. All these novel treatment protocols emphasize seed cells, or scaffolds for biomechanics, but ignore the effection of various intracorporal environment. To date, the best approach for precollapse steroid-induced ONFH remains unknown.

The current study aimed to evaluate the influence of the core decompression surgery in local marrow cell metabolism and bone regeneration and reconstruction in the steroid-associated femoral head osteonecrosis, and further explored the effects of core decompression surgery alone on the early treatment of steroid-induced osteonecrosis.

## Methods

### Animal, treatment and grouping

Forty six New Zealand healthy adult white rabbits were purchased from Laboratory Animal Center at Xi'an Jiaotong University, and were housed in according to the guidelines for the care and the use of laboratory animals delivered by the Science and Technology Committee of Xian Jaotong University. Half animals were male and other half were female, weighing from 2.6 to 3.2 kilograms (an average of 2.9 kilograms). After one week feeding adaptation, the animals were accurately weighed and randomly divided into normal control group (6 cases) and the experimental group (40 animals). There were no statistically significant differences of the animal weight and other clinical features such as gender between the two groups (p > 0.05).

### The establishment of the steroid-induced femoral head osteonecrosis rabbit model

The rabbit steroid-induced femoral head osteonecrosis model was established by the administration of steroid in combination of horse serum. Briefly, the rabbits in the experimental group were first given first injection of 10 ml/kg of horse serum (Beijing Yuan Heng Shengma Bio Institute of Technology) intravenously. After interval of 3 weeks, the second injection of horse serum was repeated, and its dose was reduced to 6 ml/kg. Two weeks after this, the animals were given an intraperitoneal injection of methylprednisolone at 45 mg/kg (Pfizer, USA) for consecutive three days. Starting from the day of hormone injection, each animal was also intraperitoneally injected with penicillin 10 million units per day for consecutive 7 days to prevent the animals from infection. The six rabbits that were injected with saline without receiving steroids were served as normal controls.

### MRI screening for osteonecrosis

In the two weeks after the last injection of hormones, the two sides of hips of all survived experimental animals were scanned with MRI. The both marrow of femoral head exhibited fat appearance such as high signal T1WI, or medium signal T2WI or low signal T1W2/SPIR (T2WI pressure fat), which was identified as potential femoral head osteonecrosis. The animals with these signals were recruited into the experimental group. Rabbits with bilateral normal femoral head marrow were classified into the normal control group. The rabbits with only one side of abnormal femoral head were removed from the study.

### Core decompression surgery and collection of bone tissue samples

The animals selected for the core decompression surgery were first weighed, and then anesthetized with a combination of valium with ketamine (15 minutes after intramuscular injection of 3.5 mg/kg of valium, ketamine was intramuscularly injected at 25 mg/kg). After anesthesia, a sterile needle for anesthesia was inserted from the flare of the greater trochanter into the femoral neck and head until about 2 mm under the greater trochanter, following the femoral shaft axis at about 45°-55° angle. The inserted needle length was not more than 2.5 cm inside the femoral marrow cavity. After successful puncture, the anesthesia needle was removed. Post-operative animals were intraperitoneally injected with 80 million units of penicillin per day for continuous three days just to prevent infection.

Tissue samples were collected at 2, 4, 8 weeks post-operation from 2 normal control animals and 5 randomly chosen surgical animals per each time point. The same anesthetic method as above was used for the specimen collection. Then bilateral femoral heads were quickly removed into prepared DEPC-treated PBS solution. After washing two times with DEPC-treated PBS buffer, half of the femoral head was immediately frozen in liquid nitrogen for future PCR and western blot analysis. The rest part was placed in 10 % paraformaldehyde solution for histological analysis.

### RNA extraction and quantitative RT-PCR

The samples of femoral head tissues from liquid nitrogen tank were mortared into powder under a sterile condition. Then they were transferred to 1.5 ml Eppendorf centrifuge tubes. The total RNA of rabbit femoral head was extracted with Trizol reagents (Invitrogen, USA). The procedures were performed strictly according to the manufacturer’s instructions. The primers for RT-PCR were designed by Oligo 6.0 primer design software (Table 1), and synthesized from Bioko synthesis (Beijing Biotech Co., Ltd.). The real-time PCR was performed by using SYBR Green PCR Kit according to the manufacturer’s instructions. The real-time PCR data was collected by an ABI 7000 Prism sequence detection system software to calculate the Ct value of all samples. The relative quantification of the gene expression in the experimental group and control group were calculated by the formulation 2^-ΔΔCt^.

**Table 1:** Primer sequences of BMP-2, PPAR-γ and GAPDH for RT-PCR

**Gene**	**Primer sequences**	**Length(bp) of product**	**Tm(°C)**	**Nucleotide number**
BMP2	5' CGA AAC ACA AAC AGC GGA AAC 3'	97	84.2	AF041421
	5' GCC ACA ATC CAG TCG TTC CA 3'			
PPARγ	5' AGT CGC CAT CCG CAT CTT 3'	147	86.8	AF013266
	5' ATC TCA TGG ACG CCG TAC TTG 3'			
GAPDH	5' TGA CAA CGA ATT TGG CTA CAG 3'	83	83.6	L23961
	5' GGT GGT TTG AGG GCT CTT ACT 3'			

According to quantitative calculation of RT-PCR (2^-ΔΔCt^), the expression of BMP-2 mRNA in the normal group at each time point was set up as 1, where ΔΔCt = ΔCt_1_-ΔCt_2_, ΔCt_1_ is a Ct value of the housekeeping gene GAPDH, ΔCt_2_ indicated the Ct value of the target gene. The level of BMP-2 and PPAR -γ mRNA expression in lateral femoral head (with or without decompression) from the normal group and operation group in the real-time RT-PCR results below was calculated by the method described above. The results were presented by group as X ± SD (n = 5).

### Western blot analysis

Proteins from rabbit femoral head tissues were isolated with RIPA lysis buffer. Total 60 μg proteins were loaded for electrophoresis on SDS-polyacrylamide gel. Then, the proteins were transferred to PVDF membranes. Rabbit anti-PPAR-γ and anti-BMP-2 (Boster Biological Engineering Co., Ltd) antibodies were diluted at a concentration of 1:500. The working concentration of internal control GAPDH antibody was 1:5000. The antibodies were incubated at 4 °C for overnight. Secondary antibody horseradish peroxidase labeled goat anti-rabbit IgG was diluted at 1:500, and incubated at room temperature for 2 h. The protein bands were visualized by DAB staining. The rabbit SP detection kit was purchased from Zhongshan Golden Bridge (SP29001). The imagines were analyzed with Bio-RAD's Quantitive one analysis software to calculate the intensity of the bands. The ratio of the intensities of the target genes and GAPDH bands was used to represent the level of the target gene protein expression.

### Histological analysis of bone tissue

Histological analyses were applied to assess the microscopic changes in the bone tissue. Prior to hematoxylin–eosin (H&E), all samples of bone tissues were fixed in 10 % (volume fraction) neutral formaldehyde solution (0. 1 mol/L, p H = 7. 4) for 3 days. Then, the specimens were decalcified in 10 % (volume fraction) EDTA-Tris buffer. The decalcification solution was replaced every week until to observe the transparent surface of the bone tissue. After decalcification was completely finished, the resultant samples were embedded by paraffin wax, and 5 μm sections were prepared. Sections were routinely stained with H&E and analysed with a microscope at various magnifications.

### Statistical analysis

All quantitative data were expressed as mean and standard error (X ± SD). Student’s *t*-test was used to examine the significance of the difference of the data between the two groups. SPSS version13.0 was used for this statistical analysis. p <0.05 indicates significant difference.

### Source of funding

This study was supported by Interdisciplinary Research Fund in Xi'an Jiaotong University (No. 8143027), Scientific and Technological Project in Shaanxi Province, and 49th China Postdoctoral Science Fund. These funding sources did not play a role in this study.

## Results

### The survive rate of animals

Five animals from the experimental group (total 40 rabbits) died during the generating the rabbit femoral head osteonecrosis model (two of which died the day after the second injection of horse serum). Two weeks after the final hormone injection, total 15 of 35 survived animals displayed early MRI features of bilateral femoral head osteonecrosis, which were recruited into the surgical group. All those15 animals remained live after the lateral core decompression operation. All 6 animals from the normal group examined by MRI showed normal bilateral femoral head.

### The expression of BMP-2 mRNA in local osteonecrosis of femoral head

**Figure 1:**
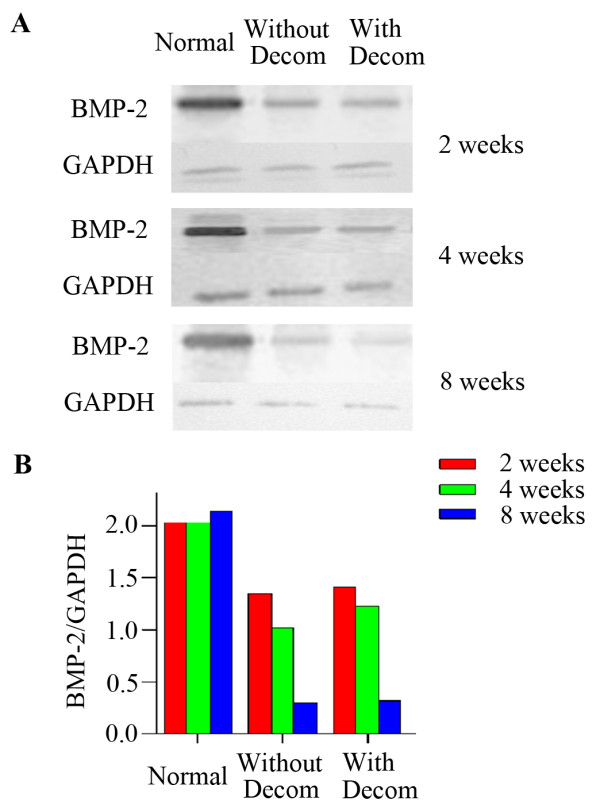
**BMP-2 protein expression in the femoral head tissue by western blot analysis.** (**A**) Western blot showed the expression levels of BMP-2 protein in the femoral head bone tissues in the normal control animals and experimental animals with or without decompression at each 2, 4 and 8 week time point; (**B**) Western blot results of BMP-2 expression in the femoral head was indicated by the ratio of band intensity of BMP-2 with GAPDH.

The local expression of BMP-2 in femoral head marrow tissue in both normal control and experimental groups was first examined by real-time PCR at each time point after decompression operation (2, 4 and 8 weeks of post-operation). As the results were shown in the Table 2, the levels of BMP-2 mRNA expression in the steroid-induced femoral head osteonecrosis with or without lateral decompression were clearly lower than that in normal group. The difference of BMP-2 mRNA expression between the experimental and normal groups was statistically significant (P < 0.05). In the experimental group, BMP-2 mRNA expression in femoral head osteonecrosis both with and without lateral decompression showed a decreasing trend with the increased time of post-operation. However, there was no significant difference of the BMP-2 mRNA expression between the femoral head osteonecrosis with and without lateral decompression, suggesting that the lateral decompression did not affect the local expression of BMP-2 in the femoral head osteonecrosis at the mRNA level (Figure 1A).

**Table 2:** BMP-2 mRNA expression in the femoral head (X ± SD, n = 5)

**Time point**	**Without decompresssion**	**With decompression**
2 weeks	0.326 ± 0.012	0.334 ± 0.032
4 weeks	0.131 ± 0.022	0.142 ± 0.021
8 weeks	0.125 ± 0.014	0.114 ± 0.011

### The expression of BMP-2 protein in local osteonecrosis of femoral head

The expression pattern of BMP-2 in femoral head from normal and experimental groups was further confirmed by the western blot at each time point of post-operation (2, 4 and 8 weeks). As can be seen in the Figure 1A and B, there was dramatically less expression of BMP-2 protein of femoral head tissue both in the steroid-induced osteonecrosis with and without lateral decompression than that in the normal control animals by western blot. In the normal group animals, BMP-2 protein expression in the femoral head tissue did not exhibit much significant difference as the post-operation time increased. In contrast, in the experimental group, the expression of BMP-2 protein in the steroid-induced femoral head osteonecrosis with and without lateral decompression both showed a progressively decreasing trend with the increased post-operation time. However, there was no significant difference of BMP-2 protein expression between femoral head osteonecrosis with and without lateral decompression, suggesting that the core decompression surgery didn’t impact the local expression of BMP-2 in the femoral head osteonecrosis at the protein level (Figure 1B).

### The expression of PPAR-γ mRNA in local femoral head osteonecrosis

**Figure 2:**
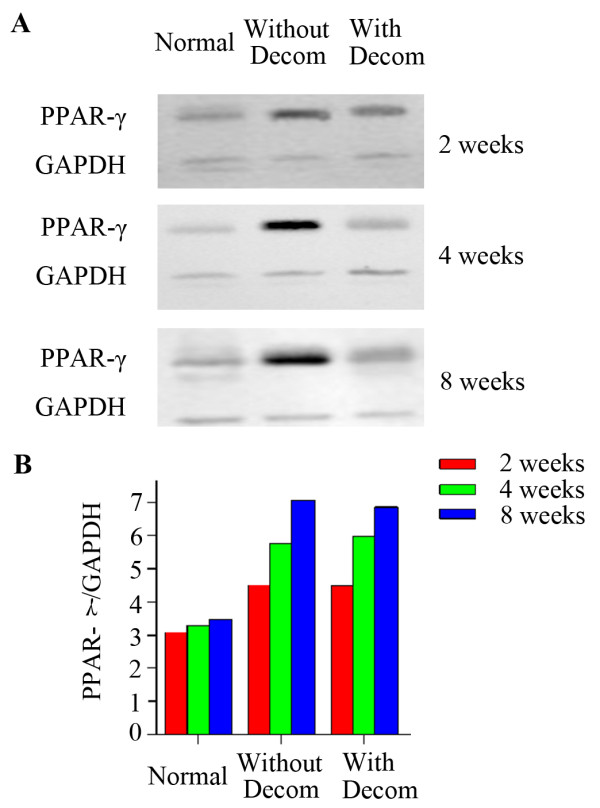
**PPAR-γ protein expression in the femoral head tissue by western blot analysis.** (**A**) Western blot showed the expression levels of PPAR-γ protein in the femoral head bone tissues in the normal control animals and experimental animals with or without decompression at each 2, 4 and 8 week time point; (**B**) Western blot results of PPAR-γ expression in the femoral head was indicated by the ratio of band intensity of PPAR-γ with GAPDH.

The expression of PPAR-γ mRNA in local femoral head was also detected by real-time RT-PCR in both normal and experimental groups at different time points of post-operation. As the results were shown in the Table 3, the expression levels of PPAR-γ mRNA both in the steroid-induced femoral head osteonecrosis with and without decompression were significantly higher than that in the control animals at all three time points of post-operation (P < 0.05). And both the expression levels of PPAR-γ mRNA in the steroid-induced femoral head osteonecrosis with and without decompression were increasing with the time of post-operation. However, there was no significant difference of the expression levels of PPAR-γ mRNA between the femoral head osteonecrosis with and without decompression, suggesting that the core decompression surgery didn’t have effect on the expression of PPAR-γ in femoral head osteonecrosis at the mRNA level (Figure 2A).

**Table 3:** PPAR-γ mRNA expression in the femoral head (X ± SD, n = 5)

**Time**	**Without decompression**	**With decompression**
2 weeks	1.258 ± 0.032	1.245 ± 0.042
4 weeks	1.432 ± 0.041	1.710 ± 0.051
8 weeks	2.013 ± 0.024	1.978 ± 0.046

### The expression of PPAR-γ protein in local femoral head osteonecrosis

We further confirmed the expression pattern of PPAR-γ in femoral head from normal and experimental groups by western blot at different time points of post-operation. As you can see in the Figure 2A and B, there was dramatically higher expression of PPAR-γ protein in femoral heads both in the steroid-induced osteonecrosis with or without lateral decompression than that in the control animals. In the normal group, PPAR-γ protein expression in the femoral head slightly increased with the increased post-operation time, but not significantly. In contrast, the expression of PPAR-γ protein in the steroid-induced femoral head osteonecrosis with and without lateral decompression both showed a significantly increased trend with the increased post-operation time. However, there was no significant difference of PPAR-γ protein expression between the femoral head osteonecrosis with and without lateral decompression at each time point of post-operation, suggesting that the decompression surgery didn’t affect the expression of PPAR-γ in the local femoral head osteonecrosis at the protein level (Figure 2B).

### The bone regeneration in the early treatment of the steroid-induced femoral head osteonecrosis

**Figure 3:**
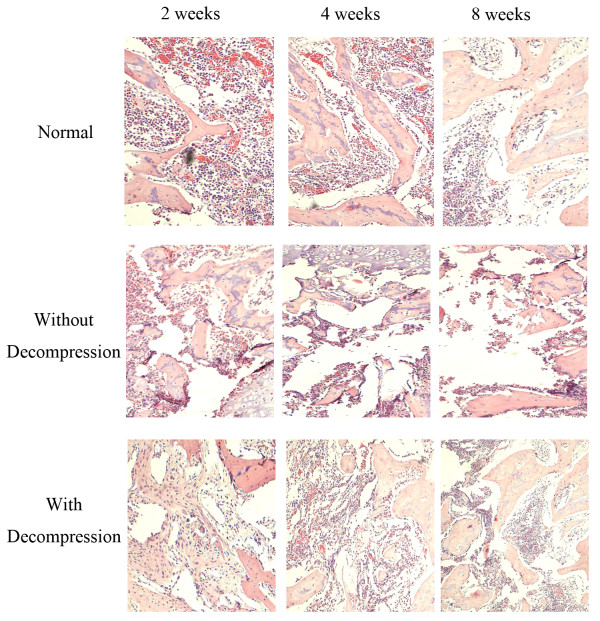
Histological analysis of the femoral head in normal control and experimental groups with or without core decompression at different post-operation time points.

We next investigated how the core decompression surgery affected the bone regeneration in the early treatment of rabbit femoral head osteonecrosis by comparing the histopathological changes of femoral head marrow tissue with or without decompression to the normal control animals at each time point of post-operation. In the normal control group, trabecular bone was continuing to exist normally at 2, 4 and 8 weeks of post-operation, intramedullary cells were normal, and no empty lacunae cells were observed. However, in the experimental group without lateral decompression, the pathogenesis of the femoral head osteonecrosis was continuing to proceed at all time points of post-operation. The trabecular bone fractures were observed and the architecture of marrow tissue was disordered. At 8 weeks of post-operation, a large number of bone marrow cell necrosis was observed and the normal bone marrow architecture was completely disappeared. In the experimental animals with decompression, decompression tunnel was seen in the femoral head osteonecrosis. In contrast, a large number of proliferative fibroblast cells were seen surrounding and filling the decompression canal at 2 weeks of post-operation. A small amount of new capillaries and ossification center started to be visible around the decompression tunnel at 4 weeks of post-operation. At 8 weeks after surgery, decompression animals showed disordered new trabecular bone and a large number of osteoblasts surrounding the decompression tunnel, but there still existed the phenomenon of trabecular bone fractures and marrow cell necrosis (Figure 3).

## Discussion

Recent studies have shown that glucocorticoid can activate the proteins of promoting the binding of fat transcription factors with enhancers (C/EBPδ, CCAAT/enhancer-binding proteins). C/EBPδ protein can specifically bind to the locus of PPAR-γ promoter and stimulate the expression of PPAR-γ gene. This would promote bone marrow stromal cells to differentiate into fat cells [12], which eventually results in the fat accumulation in the bone marrow. Yang et al. has shown that the biological effects of endogenous BMP-2 were stronger than those of purified recombinant BMP-2[13]. The BMP-2 protein generally stimulates the proliferation and differentiation of local raw bone precursor cells in autocrine and paracrine manners. And it forms a positive feedback mechanism to regulate cellular activities, which accelerates bone formation and bone repair. By in vivo and in vitro studies, Kugimiya et al. found that endogenous BMP-2 and BMP-6 cooperatively play pivotal roles in bone formation under both physiological and pathological conditions through a way of endochondral osteogenesis rather than intramembranous osteogenesis [14]. Kloen et al. also studied the local expression of BMP-2, BMP-4, and BMP-7 in twenty-one patients with a delayed union or a nonunion fracture by immunohistochemical analysis [15]. Their results showed that endogenous BMPs were continuing to present during the process of the bone regeneration and play important roles in bone repair. However, many studies have demonstrated that the local expression of endogenous BMP-2 in femoral head tissue was inhibited in the steroid-induced osteonecrosis of femoral head (GONFH), which leaded to significantly reduced functions of the MSCs in lesions, bone repair and reconstruction capacity, lack of timely repair of bone necrosis, and subchondral bone burden and mechanical dysfunction, and eventually contribute to in the femoral head collapse. Our previous studies also obtained similar results [16]. At the time that GONFH was induced, BMP-2 mRNA expression was significantly reduced compared to that in normal animals. The expression levels of BMP-2 mRNA at 2, 4 and 8 weeks post-operation were 0.326, 0.131 and 0.125 times less than that in the normal BMP-2 mRNA expression level at each time point above, respectively. In the experimental animals, BMP-2 mRNA expression showed a decreasing trend with the increased time. BMP-2 protein expression in the experimental group also exhibited the same pattern as BMP-2 mRNA in comparison with the normal control animals. By immunohistochemical staining with anti-BMP-2 antibody, BMP-2 was observed weak expression in bone cells in experimental group animals at two week time point. And BMP-2 immunostaining started to be negative at 4 week time point. In contrast, PPAR-γ mRNA was overexpressed in the femoral head in GONFH. The expression levels of PPAR-γ mRNA in the femoral medullary cavity of the experimental animals at 2, 4 and 8 week time points were 1.258, 1.432 and 2.013 times higher than that in the normal group at each time point, respectively, and progressively increased as the time extended. In the same principle, PPAR-γ protein expression in each time point was much higher than that the normal control group. In the normal control animals, it was noted that the expression levels of PPAR-γ protein were also slowly increasing with time growth. This is probably due to the physiologically accumulated fat replacement of bone marrow in the aged rabbits. Our immunohistochemistical data further confirmed that PPAR-γ expression was mainly located in the medullary cavity of the bone marrow. PPAR-γ staining in the femoral head marrow of experimental animals progressively increased to be strongly positive as the post-operation time up to 8 weeks. Therefore, the overexpression of PPAR-γ in the cells of marrow cavity in GONFH enhanced the MSCs to differentiate into fat cells, which accordingly reduce the bone cell differentiation. This contributes to a series of pathological reactions such as the fat replacement of bone marrow, the intramedullary hyperpressure, dysfunction of local microcirculation, fat cell hypertrophy and hyperplasia, localized fat thrombosis, lack of bone regeneration, and the reduction of the number and activity of osteoblast cells [17,18]. This eventually leads to the femoral head necrosis, which is one of the crucial reasons for the development of GONFH.

## Conclusions

In summary, long-term or heavy use of steroids led to over expression of PPAR-γ in the femoral head. This contributed to generation of a large number of fat cells in the bone marrow, fat replacement of bone marrow, the intramedullary hyperpressure, dysfunction of the local microcirculation, insufficient differentiation of bone cells, and reduction of the number and activities of osteoblasts [19]. Meanwhile, BMP-2 expression was inhibited, which would reduce the activities of local MSCs, and capacity of bone repair and reconstruction. Both pathological changes, on the one hand, were caused by a large number of bone cell degeneration and necrosis, and insufficient bone regeneration. On the other hand, the obstruction of reconstruction of bone tissue and slow down of bone repair eventually leaded to the osteonecrosis of femoral head, which is a major cause of morbidity of hip collapse.

The core decompression surgery is selected for the early treatment of femoral head osteonecrosis due to the simplicity of its procedure, an easy to perform operation, and less patient injury, which is easy to be acceptable for patients. Moreover, there were studies reported that the bone marrow drilling core decompression had better effects for patients with Ficat stage 0 and 1, can prevent the femoral head collapse [20]. However, mixed results about the effect of drilling decompression surgery for osteoncerosis were also reported [21,22].

Some studies even believed that core decompression was not able to improve the process of osteonecrosis. This phenomenon may be closely associated with causes which lead to femoral head necrosis. Particularly like steroid-induced osteonecrosis of femoral head, core decompression appears to be able to reduce the intramedullary pressure, improve intramedullary microcirculation, and temporarily ease the process of osteonecrosis. However, the risk factors that lead to the pathological processes of bone necrosis such as the formation of increased intramedullary pressure, dysfunction of angiogenesis in the femoral head necrosis lesion, declined capacity of bone repair and regeneration, and increased activity of osteoclast still exist. The core decompression operation alone did not directly address these series of pathological changes, because the osteonecrosis caused by application of glucocorticoids is due to the outcome of interactions between factors such as primary diseases, drugs, organ's metabolism and endocrine. In fact, GONFH is one of the side effects produced by the drug treatments which result in the metabolic and endocrine disorders in bone tissue. Thus, the effect of relying on single core decompression operation to regulate the endocrine and bone metabolism may be limited. The current studies displayed that sole decompression operation did not reverse the process of the steroid-induced osteonecrosis of femoral head, and not alter the pathological changes caused by the local decreased BMP-2 and progressively increased PPAR-γ expression. Moreover, later stage pathological examination revealed that the intramedullary bone tissue in the femoral head with lateral decompression was, despite noted the bone repair, but still visible of trabecular bone fractures and necrosis of bone marrow cells eight weeks later post-operation. The long-term effect of decompression in the bone regeneration of steroid-induced osteonecrosis is not ideal. Therefore, the basic core decompression operation in combination with other treatments like active biological molecules or local application of drugs may be able to achieve the goals of decreasing the pressure of local bone lesions as well as improving or even reversing the organism's metabolic and endocrine disorders[23-25]. Taken together, core depression surgery in combination with other local application of drugs is worth to be further investigated for the early and effective treatments of steroid-induced femoral head osteonecrosis.

## Competing interest

The author(s) declare that they have no competing interests.

## Authors’ contributions

WW, LL, and SM carried the experiments. XD, MZ did the data analysis. WW and KW designed the experiments and wrote the manuscript. All authors read and approved the final manuscript.

## Pre-publication history

The pre-publication history for this paper can be accessed here:

http://www.biomedcentral.com/1471-2474/13/142/prepub
